# Proteasome Inhibitors against Glioblastoma—Overview of Molecular Mechanisms of Cytotoxicity, Progress in Clinical Trials, and Perspective for Use in Personalized Medicine

**DOI:** 10.3390/curroncol30110702

**Published:** 2023-11-02

**Authors:** Agata Gozdz

**Affiliations:** Department of Histology and Embryology, Centre for Biostructure Research, Medical University of Warsaw, 02-004 Warsaw, Poland; agozdz@wum.edu.pl

**Keywords:** GBM, proteasome, marizomib, bortezomib, clinical trial, *MGMT*, *TP53*, *PTEN*, cell cycle, apoptosis

## Abstract

Proteasome inhibitors are moieties targeting the proteolytic activity of a proteasome, with demonstrated efficacy in certain hematological malignancies and candidate drugs in other types of cancer, including glioblastoma (GBM). They disturb the levels of proteasome-regulated proteins and lead to the cell cycle inhibition and apoptosis of GBM cells. The accumulation of cell cycle inhibitors p21 and p27, and decreased levels of prosurvival molecules NFKB, survivin, and MGMT, underlie proteasome inhibitors’ cytotoxicity when used alone or in combination with the anti-GBM cytostatic drug temozolomide (TMZ). The evidence gathered in preclinical studies substantiated the design of clinical trials that employed the two most promising proteasome inhibitors, bortezomib and marizomib. The drug safety profile, maximum tolerated dose, and interaction with other drugs were initially evaluated, mainly in recurrent GBM patients. A phase III study on newly diagnosed GBM patients who received marizomib as an adjuvant to the Stupp protocol was designed and completed in 2021, with the Stupp protocol receiving patients as a parallel control arm. The data from this phase III study indicate that marizomib does not improve the PFS and OS of GBM patients; however, further analysis of the genetic and epigenetic background of each patient tumor may shed some light on the sensitivity of individual patients to proteasome inhibition. The mutational and epigenetic makeup of GBM cells, like genetic alterations to *TP53* and *PTEN*, or *MGMT* promoter methylation levels may actually determine the response to proteasome inhibition.

## 1. Introduction

Glioblastoma (GBM, World Health Organization 4th grade *IDH1/2* wild-type glioma) is one of the deadliest malignancies in the adult population [1]. The most effective treatment comprising tumor resection, radiotherapy, and chemotherapy extends the patient’s lifetime up to 12–18 months post-diagnosis. Less than 10% of patients survive beyond five years [2]. Therefore, new treatment options capable of halting disease progression and prolonging patient life are urgently needed. Proteasome inhibitors, successfully applied as anti-leukemic drugs, seemed to be promising anti-GBM compounds. 

**Figure 1:**
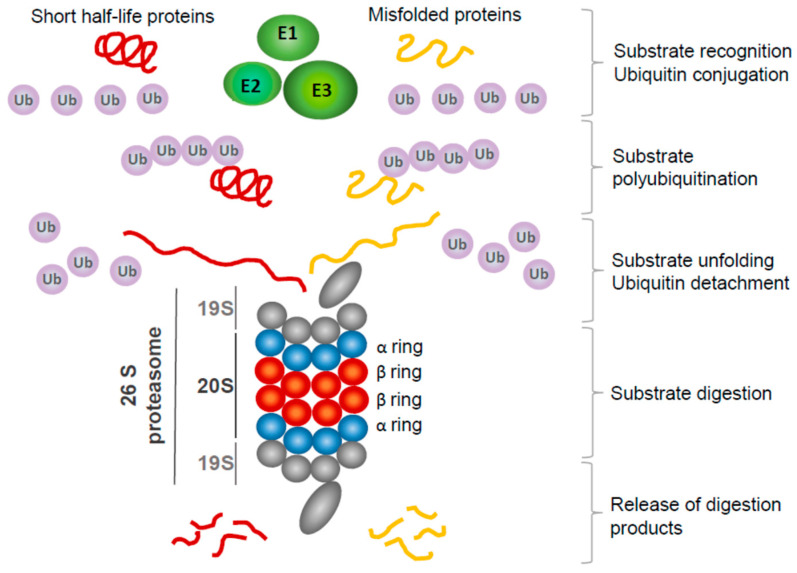
Overview of ubiquitination—proteasome system action. Most short-lived cytoplasmic and nuclear proteins, and improperly folded and dysfunctional proteins, are destined for proteasomal digestion. First, they are recognized by specific ubiquitin E3 ligase, and then E2 enzymes conjugate ubiquitin molecules activated by E1 enzymes, forming polyubiquitin chains. Polyubiquitinated proteins are targeted to the 19S subunit of a proteasome. Ubiquitin molecules are detached from protein and may be reused for the next round of polyubiquitination. Substrate protein is unfolded and directed to the 20S subunit, where β-ring proteases cleave it to short, few-amino-acids-long peptides, subsequently released from the proteasome.

The proteasome is a multiprotein complex responsible for protein degradation and the maintenance of cellular homeostasis. Proteins degraded by proteasomes require previous polyubiquitination via the ubiquitination machinery of the cell, and the coordinated activity of ubiquitination and proteasome-mediated proteolysis is defined as a ubiquitin–proteasome system (UPS) [3]. Most cytoplasmic and nuclear proteins are ubiquitinated and destroyed by the UPS. UPS action is crucial for the regulation of the lifespan of short-living substrates like cyclins or transcription factors, which fulfilled their physiological function and must be switched off through degradation, but the equally important function of UPS is the degradation of damaged or improperly folded proteins [4] (Figure 1). The activity of several UPS components is dysregulated in cancer [5]. Furthermore, many oncoproteins are UPS substrates, including those involved in cell cycle control or apoptosis [5]. The role of particular UPS-related proteins important for glioma progression and resistance to existing therapies has been recently reviewed by Maksoud [6]. The short interfering RNA (siRNA) screen employing 16,650 siRNA molecules revealed that 22% of genes identified as crucial for glioma cell line survival coded for 20S and 26S proteasome subunits [7]. These results were subsequently validated in a panel of GBM cell lines. The reported effect substantiates the use of proteasome inhibitors as potential anti-GBM drugs.

## 2. Molecular Mechanisms Underlying Proteasome Inhibitor Toxicity in GBM Cells

### 2.1. Proteasome Inhibitors Used in GBM Research

**Figure 2:**
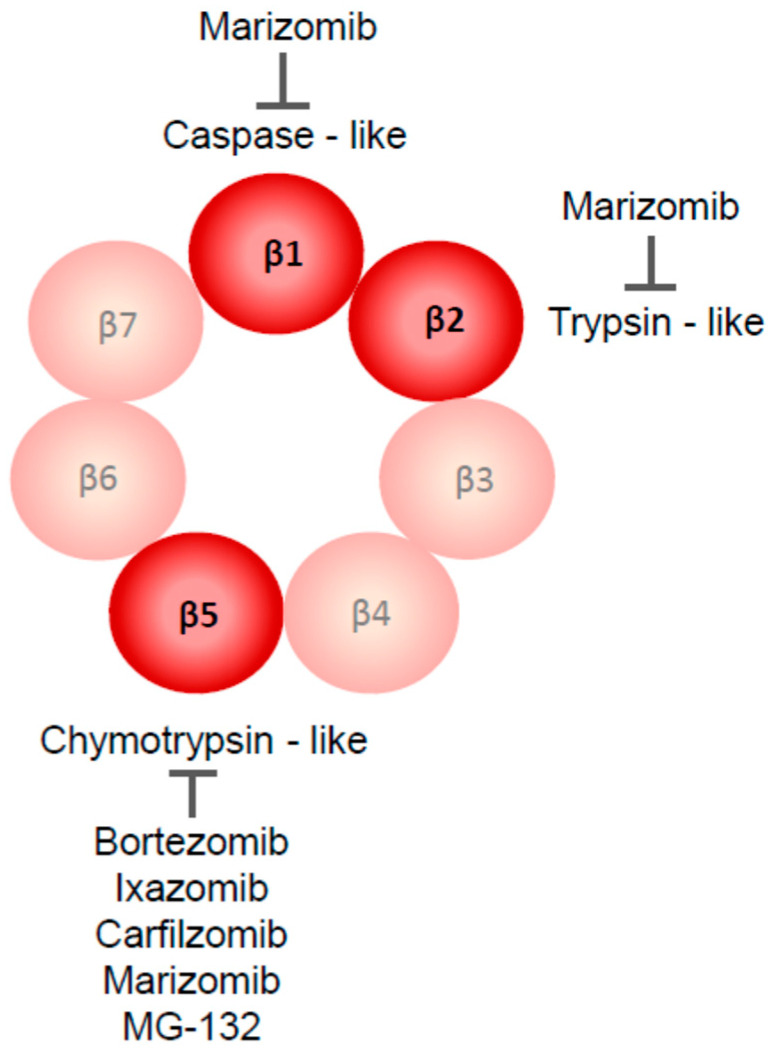
Proteolytic enzymes targeted by currently used proteasome inhibitors are parts of the β-ring of 20S proteasome. Most of them inhibit the chymotrypsin-like activity of the β5 subunit; caspase-like (β1) and trypsin-like (β2) activities are blocked only by marizomib.

Proteasome inhibitors block the enzymatic activity of the proteasome, targeting the proteolytic activity of 20S subunits (Figure 2). The inhibitors differ in structure, specificity toward proteasome enzymes, and biodistribution, thus determining their potential as anti-GBM drugs. MG-132 is broadly used in in vitro studies on proteasome function, but its metabolic stability and specificity toward proteasome enzymes are lower than that of the later-generation inhibitors [8,9]. Bortezomib and marizomib are proteasome inhibitors that were most extensively studied in various GBM in vitro and in vivo models, and their efficacy was tested in clinical trials in GBM patients. Bortezomib (PS-341, Velcade) is a boronate peptide, reversibly blocking the chymotrypsin-like activity of the 20S proteasome. The Β-lactone marizomib (Salinosporamide A, NPI—0052) irreversibly blocks three different β subunits of 20S proteasome, displaying caspase-like, chymotrypsin-like, and trypsin-like proteolytic activities, which hypothetically minimizes the possibility of the emergence of cell clones resistant to marizomib compared with other inhibitors [8,10]. Bortezomib was approved by the FDA (Food and Drug Administration) for its use in humans in 2003, initially as a drug against multiple myeloma, and later on against mantle cell lymphoma [11]. Among other proteasome inhibitors used in GBM research are carfilzomib and ixazomib, both approved to be used in humans with hematologic malignancies, targeting the primarily chymotrypsin-like activity of 20S proteasome [8,11]. Comprehensive reviews on the discovery, chemical structure, and mechanism of action of proteasome inhibitors have been published recently [8,10]. 

At the cellular level, the pharmacological inhibition or genetic ablation of proteasome activity has two main consequences, reflecting the main functions of the proteasome. First, proteasome inhibition results in disproportionate levels of certain proteins normally regulated by proteasome-mediated degradation, thus disturbing cellular functions, with various consequences for cell survival, proliferation, differentiation, and function. Second, blocking proteasome promotes the accumulation of misfolded proteins, the activation of stress response pathways, e.g., the unfolded protein response (UPR), and, when cellular stress cannot be resolved using alternative routes for the clearance of protein aggregates, cell death. 

### 2.2. Proteasome Inhibition Leads to Cell Cycle Arrest in GBM Models

Multiple cell cycle regulators are UPS substrates, including cyclins required for cell cycle progression, E2F transcription factors necessary for the onset of the S phase of the cell cycle, but also tumor suppressor p53 and cell cycle inhibitors p21 and p27 [12,13]. Bortezomib, at nanomolar concentrations, induced the accumulation of p21 and p27 proteins and cell cycle arrest at the G2/M phase in stable GBM cells and GBM explants [14]. Similarly, MG-132 treatment increased p21 protein levels in GBM cells and locked them at the G2/M phase [15]. On the other hand, Tang et al. demonstrated GBM cell cycle arrest at the G1 phase upon bortezomib application [16]. Cell cycle arrest was also shown in patient-derived GBM cell lines exposed to bortezomib [17]. Furthermore, marizomib was shown to promote the accumulation of p21 and p27 proteins in orthotopic GBM tumors in mice [18]. However, one should be aware that the increased expression of negative cell cycle regulators may result from various cellular stressors [19,20], including disturbed proteostasis upon exposure to proteasome blockers. Thus, the antiproliferative effect of proteasome inhibition in GBM cells may stem both from the simple accumulation of UPS substrates and the stimulation of stress response mechanisms. 

### 2.3. Mechanisms of Proteasome Inhibitors Triggered GBM Cell Death

**Figure 3:**
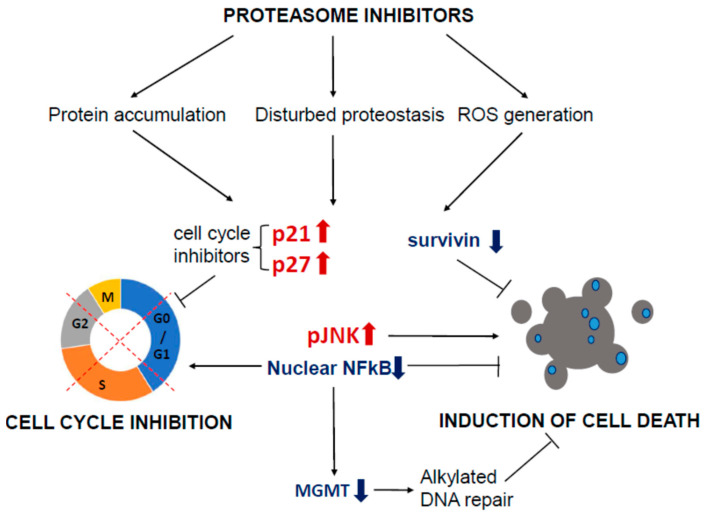
Mechanisms of action of proteasome inhibitors in GBM cells. Proteasome inhibitors induce the accumulation of proteins that fulfilled their function and would be removed from the cell in physiological conditions. Excessive accumulation of unneeded or damaged proteins leads to cellular stress and the onset of stress responses, like UPR (Unfolded Protein Response). The generation of ROS drives further damage to cellular components. The accumulation of cell cycle inhibitors, p21 and p27, and cell cycle arrest occurs in affected cells. Proteasome inhibition leads to the reduction in NF B-dependent transcription, considered a prosurvival mechanism in cancer cells. GBM cells treated concomitantly with proteasome inhibitors and TMZ display lower levels of MGMT protein. Reduced levels of anti-apoptotic proteins, like survivin of Bcl-2, are observed, and the consequence of all described processes is cell death, characterized as a caspase-dependent apoptotic cell death.

In vitro studies demonstrated proteasome inhibitors’ potential to trigger caspase-dependent apoptosis in various GBM models, including stable GBM cell lines [14,15,16,18,21,22,23,24], GBM explants [14], patient-derived GBM cell lines [17,25,26,27], and GBM organoids [25]. The cytotoxic action of proteasome inhibitors involves the generation of ROS (reactive oxygen species) that can be counteracted by the reducing agents [17,18,21,24]. Moreover, several genes and proteins controlling the endoplasmic reticulum stress response (ER-stress) and unfolded protein response (UPR) were upregulated in GBM cell lines treated with proteasome inhibitors, including pro-apoptotic protein NOXA [27]. Additionally, diminished levels of pro-survival proteins Bcl-2 and Bcl-XL were observed in GBM cell lines exposed to bortezomib [14]. The activation of JNK, but not the p38 signaling pathway, mediated the GBM cell death induced by proteasome inhibitors [14,27]. Interestingly, the reduced activity of the signaling pathway converging on the transcription factor NFκB (Nuclear Factor κB), regarded as an anti-apoptotic and defense mechanism in the tumor cell, was only observed in certain studies on proteasome-inhibitor-treated GBM cells [14] and was not detected in others when the proteasome inhibitor was used as a single agent [27,28]. The activity of the p53 pathway, crucial for a cellular stress response, cell cycle regulation, and apoptosis execution, was also triggered by proteasome inhibitors in wild-type *TP53*-expressing GBM cell lines [17]. Finally, the downregulation of an anti-apoptotic protein expression, survivin, protected GBM cells against bortezomib toxicity [16]. The most important cellular events and putative effector proteins underlying proteasome inhibitors’ anti-GBM action are depicted in Figure 3.

### 2.4. Evaluation of Proteasome Inhibitors’ Potential in Animal GBM Models

Proteasome inhibitors were tested in animal GBM models; however, they brought mixed results when the inhibitor was administered as a single agent. Bortezomib did not affect tumor volume [26,28] or reduce tumor growth [16,27,29]. Carfilzomib prevented the growth of experimental GBM tumors originating from cells with *PTEN* deletion [25]. Similarly, tumors formed by implanted GBM cells carrying the *TP53* mutation were shown to shrink upon bortezomib application [17]. Marizomib prolonged the life of mice with orthotopically implanted GBM cells [24]. It is worth noting that orthotopic models were used in certain studies [24,25,26,28,29], whereas GBM cells were injected into the flank of experimental animals in others [16,17,27]. 

The essential functionality of any potential anti-GBM therapeutic is its capability to cross the blood–brain barrier and enter tumor mass. The high heterogeneity of brain tumor vasculature determines the final degree of drug distribution, with part of the tumor vessels being hyperpermeable and allowing sufficient drug penetration, and part of them retaining low penetrability to the systemically delivered drugs [30]. Moreover, certain areas of glioma tumors are hypo-perfused, e.g., the supply of the blood vessels is inadequate, which, in turn, creates a hypoxic tumor niche hardly available for therapeutics and playing a crucial role in the development of therapy resistance and a rise in glioma malignancy [31]. The in vivo studies evaluating the ability of intraperitoneally administered bortezomib to reach tumors, such as the direct measurement of drug concentrations or via the determination of intratumoral chymotrypsin-like protease activity, revealed that bortezomib was present within the implanted GBM tumor and the relevant enzymatic activity of proteasome was reduced [28]. Moreover, the bortezomib intratumoral level surpassed that detected in GBM patient plasma by several folds [32]. Marizomib presence and inhibitory activity within intact rat and monkey brains, respectively, were shown by Di et al. [24]. Twenty-four hours post-marizomib application, its concentration in rat brain reached around 50% of that measured in blood; marizomib administration inhibited the chymotrypsin-like and caspase-like activity of 20S proteasome by 30% in the intact brain of cynomolgus monkey [24]. The presented data suggested that both proteasome inhibitors may reach intracranial tumors and exert their cytotoxic effect on GBM tumor cells. However, Wang et al. reported that bortezomib given intravenously was effective in shrinking experimental subcutaneous glioma tumors, but not intracranial ones [33]. The volume of the latter was reduced upon the administration of bortezomib through the implanted mini-osmotic pump [33]. This report strongly implies that the proteasome inhibitor concentration and administration route are important factors during the evaluation of the anti-GBM properties of these drugs in vivo.

### 2.5. Potentiation of TMZ Toxicity by Proteasome Inhibitors

TMZ is a chemotherapeutic drug administered for GBM patients receiving radiotherapy and as an additional (adjuvant) treatment after radiotherapy completion [34] or the drug of choice for recurrent GBM for patients who responded to TMZ in the first line of treatment [35]. TMZ acts as an alkylating agent targeting guanine in DNA strands leading to its transformation to 6-methylguanine, and this altered base is removed by the mechanism involving acceptor protein, MGMT (O-6-methylguanine-DNA methyltransferase) [36]. Thus, MGMT expression is an important mechanism determining TMZ sensitivity, and the level of its expression strongly depends on the extent of *MGMT* promoter methylation [36]. Currently, *MGMT* promoter status is considered an important predictive biomarker in GBM patients, since a higher level of *MGMT* promoter methylation and a lower level of MGMT protein expression translates to better response to treatment with TMZ, and longer patient survival [37]. 

The preclinical studies demonstrated that proteasome inhibitors potentiate TMZ toxicity in GBM models. Bortezomib increased TMZ toxicity in GBM cell lines, and it was accompanied by the stabilization of the IκB protein, acting as an NFκB inhibitor, which resulted in lower levels of nuclear NFκB and MGMT protein expression [38]. Rahman et al. reported the reduced expression of MGMT upon bortezomib and TMZ co-treatment, and the synergistic action of these drugs in GBM cell lines with unmethylated MGMT promoter [28]. They also demonstrated the potential of concomitant bortezomib and TMZ to decrease tumor volume in an intracranial glioma model [28]. This observation underlies the design of the current clinical trial investigating the efficiency of bortezomib and TMZ in patients with unmethylated MGMT promoter [39]. Furthermore, Tang et al. reported that bortezomib sensitized resistant GBM cells to TMZ and potentiated the TMZ-induced shrinkage of tumors formed from subcutaneously implanted GBM cells [16]. TMZ increased the expression of anti-apoptotic protein survivin in vitro and in vivo, and bortezomib efficiently reduced survivin levels [16].

### 2.6. Synergistic Action of Proteasome Inhibitors and Other Potential Anti-GBM Drugs

It is also worth acknowledging that proteasome inhibitors may sensitize GBM cells to other cytotoxic factors. Bortezomib reduced NFκB levels in TRAIL (tumor necrosis factor-related apoptosis ligand)-resistant GBM cell lines and sensitized them to TRAIL-mediated cell killing [40]. The sensitization to TRAIL and TNFα (Tumor Necrosis Factor α)-dependent cell killing was observed in GBM cells co-treated with bortezomib [14]. Also, marizomib sensitized GBM cells to synthetic TRAIL receptor agonists [41]. TRAIL signaling was also required for the lysis of GBM cells exposed to bortezomib and cultured in the presence of NK (Natural Killer) cells [29]. Interfering with epigenetic modifier activity, like HDACs (histone deacetylases), increased the toxicity of bortezomib and marizomib in preclinical glioma studies [18]. However, patients with recurrent GBM who received the HDAC inhibitor vorinostat in combination with bortezomib show no improvement [42]. Another interesting area of research is the concomitant blockade of UPS and autophagy. Indeed, autophagy inhibitor 3-MA potentiated the toxicity of bortezomib in GBM cell lines [23]. 

### 2.7. Potential Mechanisms Impeding Proteasome Efficiency as Anti-GBM Agents

Bota et al. demonstrated an important side-effect of proteasome inhibition. Bortezomib induced the accumulation of proteasome target, hypoxia-inducible factor 1 alpha (HIF1a), and subsequent Vascular Endothelial Growth Factor A (VEGF A) synthesis in GBM cells [26]. The pro-angiogenic and pro-survival actions of VEGFA may be reduced by an anti-VEGFA antibody, bevacizumab. The combination of bevacizumab and bortezomib was slightly more potent in terms of tumor shrinking and extending mice survival than bevacizumab alone [26]. However, the combination of the BBB-permeant proteasome inhibitor, marizomib, did not augment bevacizumab’s anti-GBM effect in recurrent GBM patients [43]. 

There are also reports on other undesired effects of the treatment of GBM cells with proteasome inhibitors. Lin et al. demonstrated that bortezomib activated the pro-survival PI3K/Akt pathway in GBM cells, and this proteasome inhibitor was only able to kill GBM cells upon the simultaneous pharmacological blockade of PI3K/Akt pathway activity [44]. Next, Manton et al. reported the protective role of activated caspase 2 in GBM cells exposed to marizomib. Moreover, several cyclin levels were increased upon proteasome inhibition [14,44]. Finally, bortezomib was shown to lead to the calpain-dependent degradation of IκB and the resulting increase in NFκB levels [45].

## 3. Clinical Research on Proteasome Inhibitors as Anti-GBM Agents

If eligible, newly diagnosed GBM patients are usually treated according to the Stupp protocol, comprising tumor resection followed by radiotherapy with concomitant chemotherapy with the use of TMZ, and the adjuvant application of up to six cycles of TMZ [34]. The applied protocol is not curative, and the disease returns months post-initial surgery in an even more vicious and hard-to-eradicate form, displaying mutational, transcriptomic, proteomic, and immunological characteristics distinct from those detected in the tumor at the time of diagnosis. The treatment of recurrent GBM varies, including repeated neurosurgery, next courses of radiotherapy, and the use of genotoxic agents, e.g., TMZ, carmustine and vincristine, anti-VEGF antibody bevacizumab, and experimental drugs [46]. 

Initial studies aiming to evaluate the bortezomib and marizomib safety profile in GBM patients used either as a single drug or in combination with other therapeutic modalities were performed mostly with the participation of recurrent GBM patients (Table 1). 

### 3.1. Evaluation of Bortezomib Safety Profile and Therapeutic Efficacy in GBM Patients

Bortezomib’s pharmacokinetics, pharmacodynamics, and safety profile were defined well before any trials evaluating its anti-glioma potential [52]. However, the combination of bortezomib and drugs administered to glioma patients required additional studies to find the safe dose of investigated drugs and pre-screen the efficacy of bortezomib as a single GBM-killing agent or in association with other anti-GBM protocols. The first clinical trial in glioma patients proved the safety of bortezomib in combination with radiotherapy and the DNA-alkylating agent, TMZ, and determined that the highest safe bortezomib dose that can be used in patients treated with the investigated drug combination is 1.3 mg/m^2^ [47]. Bortezomib was administered simultaneously with radiation and TMZ [47]. Another study, performed predominantly in patients with recurrent malignant glioma, determined the maximum tolerated dose (MTD) of bortezomib in patients receiving anti-seizure drugs that may alter the activity of hepatic enzymes (EIADs, enzyme-inducing anti-seizure drugs), resulting in the increased metabolism of bortezomib [48]. Bortezomib at MTD range, defined for patients not taking EIADs (1.7 mg/m^2^), efficiently blocked 20S proteasome activity in the whole blood of treated patients [48]. However, due to the increased removal of bortezomib, individuals on EIADs required the administration of higher doses of bortezomib to obtain the same level of 20S proteasome inhibition (2.1 mg/m^2^), which was still below the MTD defined for this group [48]. Accordingly, Portnow et al. demonstrated that drugs that affected hepatic enzyme activity (HEIAs) increased bortezomib clearance in patients with various malignancies receiving bortezomib and TMZ when compared to patients who did not take HEIAs [49]. Because anti-convulsant drugs, e.g., carbamazepine, oxacarbazepine, phenytoin, or phenobarbital, may be administered to GBM patients to relieve some symptoms, one should be aware of their effect on bortezomib metabolism, and the same rule applies to other drugs used in clinical trials. 

Another drug combination whose therapeutic potential was investigated in GBM patients comprised bortezomib and the inhibitor of histone deacetylases, vorinostat. Friday et al. found that bortezomib did not potentiate the anti-glioma effect of vorinostat in patients with recurrent disease, despite the promising preclinical results [42]. The next trial with the participation of 10 recurrent glioma patients treated with bortezomib and TMZ revealed that the bortezomib concentration in the tumor was higher than in the patient’s blood, which demonstrates the drug’s ability to enter the tumor [32]. However, none of the patients receiving bortezomib with TMZ survived beyond six months, and the trial was terminated [32]. The same drug combination was applied to recurrent GBM patients with unmethylated MGMT promoter [39]. Some differences in immune cell activation were observed depending on PFS and OS length; however, the results from only 10 subjects were analyzed [39]. 

Promising results of a clinical trial evaluating bortezomib in GBM-affected patients were obtained by Kong et al. [50]. Twenty-four newly diagnosed high-grade glioma patients with defined levels of MGMT promoter methylation participated in the study. The whole group received the Stupp protocol supplemented with bortezomib concomitantly with adjuvant TMZ. The historical data from GBM patients to whom the standard Stupp protocol was applied served as a control arm. Bortezomib co-treated GBM patients with methylated MGMT promoter survived for much longer than patients treated with adjuvant TMZ only, with a median PFS of 24.7 months for the bortezomib group and 10.3/14.1 months for historical control groups from two previously published studies, and a median OS of 49.4 months and 21.7/23.2 months, respectively [50]. On the other hand, the supplementation of standard chemotherapy with bortezomib had no effect on PFS and OS in patients treated with unmethylated MGMT promoter when compared to historical data [50]. 

Grade 3 or 4 adverse events observed across the studies employing bortezomib in glioma patients comprised peripheral neuropathies, headache, fatigue, thrombocytopenia, leukopenia, and gastrointestinal effects [47,48,50]. The largest study on bortezomib [50] reported grade 3 adverse events in 25% of patients and 29% of patients in the RT-phase and post-RT phase of the protocol, respectively. Grade 2 toxicities associated with bortezomib administration resulted primarily from disturbed hemopoiesis (thrombocytopenia, lymphopenia, and neutropenia), but also included nervous-system-related effects (headache, seizures, disturbed cognition), skin irritation, and fatigue [50]. 

### 3.2. Evaluation of Marizomib Safety Profile and Efficacy in GBM Patients

The first results of clinical studies employing marizomib in GBM patients have been posted very recently. Since marizomib was used in GBM patients for the first time, the determination of the safety profile, including the maximum tolerated dose and detailed characteristics of the side effects, was of crucial importance. Bota et al. performed a study with the participation of recurrent GBM patients. The highest tolerated dose of marizomib was defined as 0.8 mg/m^2^, and the most frequently observed drug side effects included fatigue, headache, hallucinations, and insomnia [43]. Altogether, CNS-related adverse events of any grade were observed in 85% of patients receiving marizomib as a single agent and provided indirect evidence that marizomib was, indeed, present behind the blood–brain barrier. Relevant pharmacological interventions helped to manage the CNS-related side effects. Moreover, the robust and long-term inhibition of proteasome activity was demonstrated in blood from patients receiving marizomib. The second part of the study aimed to determine whether marizomib cooperated or synergized with an antibody-targeting VEGF, bevacizumab (Avastin). The rationale behind this combination originated from the observation that proteasome inhibition with bortezomib elevated the levels of VEGF in GBM stem-like cells [26]; therefore, supplementation with bevacizumab was hypothesized to ameliorate this particular undesirable marizomib effect. Bevacizumab is a drug of choice in the treatment of recurrent GBM patients, shown to improve patient PFS. Here, marizomib was added to bevacizumab and the effect of the drug combination was compared to historical data from patients treated with the same amount of bevacizumab (10 mg/kg) as a monotherapy. Adding marizomib did not improve the survival of the patients treated with both drugs when compared to historical data from patients treated with bevacizumab alone [43].

Finally, the largest clinical trial evaluating marizomib effects was conducted in 749 newly diagnosed GBM patients. Patients were allocated to one of two groups: the control group receiving surgery, standard RT, and TMZ, followed by adjuvant TMZ (Stupp protocol) and the placebo or experimental group receiving Stupp protocol and marizomib [51]. The initial analysis of the clinical trial results indicates that marizomib is not able to improve PFS (median 6.1 months in the control arm and 6.2 months in the marizomib arm) and OS (median 15.9 months in the control arm and 15.7 months in marizomib arm) of GBM patients when added to the standard protocol [51]. However, the secondary analyses, like the determination of the effects of MGMT promoter status on marizomib efficacy, are planned and may help to answer the question of whether tumor/patient features affect the response to marizomib combined with standard GBM care. There are also other I/II phase clinical trials in GBM patients, including those combining radiochemotherapy and tumor-treating fields (NCT02903069) or modified rapamycin (NCT03463265) with marizomib; however, they are still ongoing, or their results have not been published yet. 

Marizomib-associated toxicities have been observed primarily in the CNS whenever marizomib was used as a single agent, in combination with bevacizumab, or with the Stupp protocol [43,51]. The initial report from the NCT03345095 trial [51] indicated that 42.6% of patients from the marizomib arm experienced grade 3/4 adverse events compared to 20.6% of patients from the control arm receiving the Stupp protocol only. Therefore, this may suggest that, in GBM patients, marizomib is more likely to generate side effects than bortezomib, especially those that affect nervous system function. Importantly, due to the rapid evolution of the GBM research field, also based on the analysis of patient-specific responses to the treatment, the World Health Organization (WHO) has issued a new classification of glioma tumors [53]. In this review, for studies conducted before 2021, the original nomenclature is retained. One should be aware that tumors classified in the past as GBM may carry an *IDH1*/*IDH2* mutation or exhibit a G-CIMP phenotype (with or without an *IDH1*/*IDH2* mutation), both of which are prognostic factors for longer survival in glioma patients [54]. Furthermore, in the case of a small group of patients participating in phase I or II clinical trials, there is always a risk of an imbalance between study arms regarding patient background and treatment sensitivity.

## 4. Perspectives for Proteasome Inhibitors as Precision Anti-GBM Drugs

Thus far, the results of experiments searching for the correlation between the level of MGMT promoter methylation and sensitivity to proteasome inhibition do not give a clear answer as to whether MGMT promoter status could be treated as a predictor of combined therapy success, e.g., radio- and chemotherapy with concurrent proteasome inhibition [28,38,50]. Only the analysis of balanced datasets obtained during clinical tests would allow us to answer the above question.

**Figure 4:**
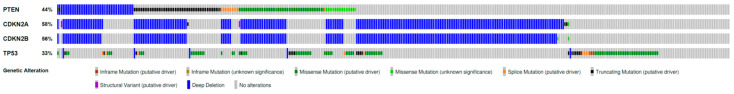
Frequency and distribution of DNA alterations in genes of interest in TCGA GBM set that putatively determine differential sensitivity to proteasome inhibition. The oncoplot shows the mutual exclusivity or co-occurrence of mutations in genes shown to affect the response to proteasome inhibitors, as demonstrated in in vitro and in vivo studies.

Recently, Johansson et al. performed a drug library screen using a large cohort of GBM patient-derived cell lines and found a correlation between *TP53* (Tumor Protein 53) and *CDKN2A/CDKN2B* (Cyclin Dependent Kinase Inhibitor 2A and 2B) mutational and functional status and cell line sensitivity to proteasome inhibitors [17]. Interestingly, cell lines harboring mutations in the *TP53* gene, but without *CDKN2A/CDKN2B* loss, were more sensitive to proteasome inhibition than cell lines without these particular alterations [17]. On the other hand, Benitez et al. demonstrated that the pharmacological proteasome blockade preferably affected cell lines with mutated/deleted *PTEN* (Phosphatase and Tensin Homolog) and the subsequent hyperactivation of the PI3K/Akt/mTOR pathway [25]. To demonstrate the distribution of alterations in *TP53*, *PTEN*, and *CDKN2A/CDKN2B* genes, an oncoplot was generated with the cBioPortal online tool [55,56], based on TCGA (The Cancer Genome Atlas, Pan-Cancer Atlas) data from 585 GBM patients and the search criteria set for any identified genetic alteration in the genes of interest. The allocation of changes in *PTEN*, *TP53,* and *CDKN2A/CDKN2B* genes in clinical samples suggests that two noticeably distinct groups of patients may benefit more from proteasome inhibition than the others (Figure 4). The high expression of functional p53 protein may also suppress MGMT levels and sensitize GBM cells to TMZ [57]. As current clinical trials assess new anti-GBM drugs in the form of adjuncts to the Stupp protocol, the mutational and functional status of p53 may influence the response to treatment, and this notion is also applicable to proteasome inhibitors. Altogether much more extensive basic research and thorough analysis of clinical data would be required to assess proteasome inhibitors’ potential as personalized anti-GBM therapeutics, e.g., drugs matching the molecular characteristics of the particular tumor.

## 5. Concluding Remarks and Future Perspectives

Proteasome inhibitors are interesting moieties characterized by extremely pleiotropic modes of action, and show some potential to be used as anti-GBM agents. Bortezomib and next-generation proteasome inhibitors greatly improved the prognosis of patients with B-cell-associated hematological malignancies. However, no response was observed in the subset of hematological cancer patients receiving proteasome inhibitors for the first time [11]. Furthermore, secondary resistance to proteasome inhibitors develops after an initial successful drug response. It cannot be excluded that the situation is similar in GBM patients, and only a fraction of these patients would benefit from adjuvant proteasome inhibition. Although bortezomib and marizomib were shown to penetrate the blood–brain barrier, it does not guarantee that cancerous cells are reached sufficiently in every tumor location. Various mechanisms of resistance may emerge in treated cells, including alternative routes for the removal of unfolded/unnecessary proteins. Recent studies suggest that the sensitivity of GBM cells to proteasome inhibitors is influenced by their mutational/epigenetic and transcriptomic/proteomic characteristics. Tailoring these inhibitors individually for each patient may be the only feasible approach to further develop them as anti-GBM agents. Additionally, omics approaches can be augmented by the drug sensitivity screening of tumor-derived organoids to determine the most effective drug or drug combination for each GBM patient.

## Figures and Tables

**Table 1:** Clinical trials testing proteasome inhibitors in GBM.

No.	Trial Phase	Number of Patients	Tumor Type/Status	Treatment Investigated	Year of the Publication	Trial #No. ^d^, Publication
1.	I	19 ^a^	Newly diagnosed and recurrent GBM ^b^	Bortezomib (dose escalation study), RT, TMZ	2009	[47]
2.	I	51 ^b^	Recurrent GBM	Bortezomib (dose escalation study), EIASDs	2010	[48]
3.	II	37	Recurrent GBM	Bortezomib, Vorinostat	2011	[42]
4.	I	25 ^c^	Solid tumors	Bortezomib, TMZ, HEIA’s	2012	[49]
5.	II	10	Recurrent GBM	Bortezomib, TMZ	2016	[32]
6.	II	24	Newly diagnosed GBM	Bortezomib, RT, TMZ	2018	NCT00998010, ref. [50]
7.	I/II	10	Recurrent GBM	Bortezomib, TMZ	2020	NCT03643549, ref. [39]
8.	I/II	30	Recurrent GBM	Marizomib (dose escalation study)	2021	NCT02330562, ref. [43]
9.	I/II	67	Recurrent GBM	Marizomib, Bevacizumab	2021	NCT02330562, ref. [43]
10.	III	749	Newly diagnosed GBM	Marizomib, RT, TMZ	2021	NCT03345095, ref. [51]

^a^ Number of patients diagnosed with GBM, including 11 patients with newly diagnosed GBM and 8 patients with recurrent GBM; ^b^ out of 66 patients enrolled in the study, 51 had a diagnosis of recurrent GBM; ^c^ out of 25 patients enrolled, 2 received a high-grade glioma diagnosis; ^d^ for earlier trials, NCT numbers are not specified.
